# Oral Scutellarin Treatment Ameliorates Retinal Thinning and Visual Deficits in Experimental Glaucoma

**DOI:** 10.3389/fmed.2021.681169

**Published:** 2021-08-03

**Authors:** Jingyuan Zhu, Anoop Sainulabdeen, Krystal Akers, Vishnu Adi, Jeffrey R. Sims, Eva Yarsky, Yi Yan, Yu Yu, Hiroshi Ishikawa, Christopher K. Leung, Gadi Wollstein, Joel S. Schuman, Wenbin Wei, Kevin C. Chan

**Affiliations:** ^1^Beijing Tongren Eye Center, Beijing Key Laboratory of Intraocular Tumor Diagnosis and Treatment, Beijing Ophthalmology & Visual Sciences Key Lab, Medical Artificial Intelligence Research and Verification Key Laboratory of the Ministry of Industry and Information Technology, Beijing Tongren Hospital, Capital Medical University, Beijing, China; ^2^Department of Ophthalmology, NYU Grossman School of Medicine, NYU Langone Health, New York University, New York, NY, United States; ^3^Department of Surgery and Radiology, College of Veterinary and Animal Sciences, Kerala Veterinary and Animal Sciences University, Thrissur, India; ^4^Pleryon Therapeutics Limited, Shenzhen, China; ^5^Department of Biomedical Engineering, Tandon School of Engineering, New York University, New York, NY, United States; ^6^Hong Kong Eye Hospital, University Eye Center, Hong Kong, China; ^7^Department of Ophthalmology and Visual Sciences, The Chinese University of Hong Kong, Hong Kong, China; ^8^Department of Ophthalmology, The University of Hong Kong, Hong Kong, China; ^9^Center for Neural Science, College of Arts and Science, New York University, New York, NY, United States; ^10^Neuroscience Institute, NYU Grossman School of Medicine, NYU Langone Health, New York University, New York, NY, United States; ^11^Department of Radiology, NYU Grossman School of Medicine, NYU Langone Health, New York University, New York, NY, United States

**Keywords:** glaucoma, intraocular pressure, scutellarin, retina, optokinetics

## Abstract

**Purpose:** Intraocular pressure (IOP) is currently the only modifiable risk factor for glaucoma, yet glaucoma can continue to progress despite controlled IOP. Thus, development of glaucoma neurotherapeutics remains an unmet need. Scutellarin is a flavonoid that can exert neuroprotective effects in the eye and brain. Here, we investigated the neurobehavioral effects of scutellarin treatment in a chronic IOP elevation model.

**Methods:** Ten adult C57BL/6J mice were unilaterally injected with an optically clear hydrogel into the anterior chamber to obstruct aqueous outflow and induce chronic IOP elevation. Eight other mice received unilateral intracameral injection of phosphate-buffered saline only. Another eight mice with hydrogel-induced unilateral chronic IOP elevation also received daily oral gavage of 300 mg/kg scutellarin. Tonometry, optical coherence tomography, and optokinetics were performed longitudinally for 4 weeks to monitor the IOP, retinal nerve fiber layer thickness, total retinal thickness, visual acuity, and contrast sensitivity of both eyes in all three groups.

**Results:** Intracameral hydrogel injection resulted in unilateral chronic IOP elevation with no significant inter-eye IOP difference between scutellarin treatment and untreated groups. Upon scutellarin treatment, the hydrogel-injected eyes showed less retinal thinning and reduced visual behavioral deficits when compared to the untreated, hydrogel-injected eyes. No significant difference in retinal thickness or optokinetic measures was found in the contralateral, non-treated eyes over time or between all groups.

**Conclusion:** Using the non-invasive measuring platform, oral scutellarin treatment appeared to preserve retinal structure and visual function upon chronic IOP elevation in mice. Scutellarin may be a novel neurotherapeutic agent for glaucoma treatment.

## Introduction

Glaucoma is a chronic neurodegenerative disease involving progressive loss of retinal ganglion cells (RGC) and injuries to their dendrites and axons (1). It is the leading cause of irreversible blindness in the world (2). While intraocular pressure (IOP) is currently the only clinically modifiable risk factor, glaucoma can continue to progress even after IOP is controlled (3). Thus, development of effective neurotherapeutics is of paramount importance to further slowdown the progression of the disease beyond IOP control in order to reduce its prevalence.

Several *in vitro* and *in vivo* experiments have attempted to demonstrate the potentials of neuroprotective medications for glaucoma. These include the use of alpha 2 adrenergic agonists (e.g., brimonidine) (4), prostaglandin-related compounds (e.g., tafluprost) (5), N-methyl-D-aspartate receptor antagonists (e.g., memantine) (6), calcium channel blockers (e.g., nilvadipine) (7), choline precursor (e.g., citicoline) (8), brain-derived neurotrophic factors (9), and plant extracts [e.g., ginkgo biloba (10), xanthophylls, and flavonoids (11)]. However, their effectiveness on glaucoma patients has been controversial (12), and the side effects of some of these medications remain a concern (13). Among the plant extracts that have been identified as potential candidates for protective effects, *Erigeron breviscapus* is a multifunctional traditional Chinese herb that has been used to treat various diseases in the brain and body of both humans and experimental animal models (14). Scutellarin, a flavone glucuronide (5,6,4′-trihydroxyflavone-7-O-glucuronide), is one of the major constituents of *Erigeron brevisca*pus (15). It has been reported to exert protective effects on the brain (16) via translocation of the apoptosis-inducing factor pathway (17), and increase in cell survival, proliferation, and contraction (18). In ophthalmic studies, scutellarin promoted the survival of cultured rat retinal neurons at high concentrations (19). Scutellarin was also found to preserve, at least in part, the visual field of post-surgical open-angle glaucoma patients with controlled IOP (20). For acute IOP elevation and retinal hypoxia models, scutellarin inhibited the inflammatory reactions by mediating the nucleotide-binding oligomerization domain-like receptor protein 3 (NLRP3) inflammasome-signaling pathway *in vivo* and *in vitro* (21). It also promoted RGC survival while down-regulating abnormal retinal microglia activation (21). Despite such initial evidence, well-controlled experiments on the neuroprotective effects of scutellarin on chronic glaucoma remain lacking.

To date, pre-clinical testing of glaucoma neurotherapeutics has been limited by existing experimental models that allow chronic elevation of IOP while preserving optical media clarity for non-invasive and longitudinal evaluation of the structure and function of the visual system. Recently, we have developed a novel experimental glaucoma rodent model that satisfies the above requirements via intracameral injection of an optically clear, cross-linked hydrogel (22, 23). In the current study, we used this experimental model in combination with non-invasive imaging and behavioral assessments to investigate if scutellarin is neuroprotective against chronic experimental glaucoma. We hypothesized that oral scutellarin treatment ameliorates the effects of retinal thinning and visual functional deficits induced by chronic IOP elevation.

## Methods

### Animal Preparation

All experimental procedures were approved by the Institutional Animal Care and Use Committee at New York University Grossman School of Medicine, and investigators followed guidelines from the Association for Research in Vision and Ophthalmology's statement for Use of Animals in Ophthalmic and Vision Research. Thirty C57BL/6J mice (Jackson Laboratory, Bar Harbor, Maine) aged 15–18 weeks old were housed under a 12-h light/dark cycle with standard chow and water available *ad libitum*, and were assigned to three groups of 10 each via a random number table. Mice in Group 1 received unilateral intracameral injection of hydrogel only (Hydrogel group). Mice in Group 2 received unilateral intracameral injection of phosphate-buffered saline (PBS) only (PBS group). Mice in Group 3 were unilaterally injected with the hydrogel into the anterior chamber along with daily oral gavage of scutellarin at 300 mg/kg/day for 3 consecutive weeks, beginning at 1 week prior to hydrogel injection until 2 weeks post-injection (Hydrogel+Scutellarin group) (Table 1). The contralateral eyes were untreated and served as an internal control. A non-invasive *in vivo* measurement system was developed for longitudinal assessments of IOP via tonometry, retinal thickness via optical coherence tomography, and visual function via optokinetic behavioral testing immediately before, and at 3 days (IOP only), and 1, 2, 3, and 4 weeks after intracameral hydrogel or PBS injection (Table 1). For Group 3, the same *in vivo* measurements were performed at an additional time point at 1 week before intracameral hydrogel injection (i.e., before daily oral scutellarin treatment began). Throughout the experiments, the researchers did not know which eye had been injected until group data analyses were to be performed.

**Table 1:** Overall experimental timetable.

**Experimental procedures/** **Time after intracameral injection**	**−1 week**	**0 week**	**3 day**	**1 week**	**2 week**	**3 week**	**4 week**
Tonometry	Group 3 only	All	All	All	All	All	All
Optical coherence tomography	Group 3 only	All	-	All	All	All	All
Optokinetics	Group 3 only	All	-	All	All	All	All
Intracameral hydrogel or PBS injection	-	All	-	-	-	-	-
Daily scutellarin treatment	Group 3 only	Group 3 only	Group 3 only	Group 3 only	Group 3 only	-	-

*-Group 1: Hydrogel-only group*.

*-Group 2: Phosphate-buffered saline (PBS) group*.

*-Group 3: Hydrogel+Scutellarin group*.

*-Sample size (N) for tonometry/optical coherence tomography/optokinetics – visual acuity (VA): Group 1 = 10; Group 2 = 8; Group 3 = 8*.

*-N for optokinetics – contrast threshold (CT): Group 1 = 9; Group 2 = 8; Group 3 = 4*.

### Intracameral Hydrogel or PBS Injection

All mice were anesthetized with an intraperitoneal injection of a 5:1 ketamine/xylazine cocktail at 0.01 mL/g body weight (Henry Schein, NY). The side for unilateral intracameral injection in each animal was selected randomly by a coin toss. Proparacaine and tropicamide were then topically applied to the randomly chosen eye to induce analgesia and pupil dilation followed by intracameral injection with 2 μL of a 1:1 mixture of vinyl sulfonated hyaluronic acid and thiolated hyaluronic acid (Groups 1 and 3) or PBS (Group 2) via a glass micropipette (BLAUBRAND^®^, BR708709-1000EA). The functionalized hyaluronic acid mixture was heat-sensitive and became gelatinous in the anterior chamber since body temperature triggered chemical crosslinking of the polymers. This solidified hydrogel obstructed the aqueous outflow, thereby causing a sustained increase in IOP with long-term preservation of a transparent optical media for at least 4 weeks. Antibiotic ointment was applied topically immediately after injection, and ophthalmic hydrogel was applied to the surface of the eye without intracameral injection. A successful intracameral injection was judged by the absence of iris penetration, lens abrasion, and other traumatic damage. One mouse in Group 1 presented hyphema during intracameral injection as a result of unintentional needle injury to the iris, and was replaced by a spare mouse of the same age in the same cohort. Two mice in Group 2 and two mice in Group 3 did not survive through the entire study period due to other concerns including needs of euthanasia from fighting wounds. These four animals were not replaced and none of their incomplete data was included in the statistical analyses.

### Intraocular Pressure Measurements Using Tonometry

The IOPs of both eyes were measured under inhaled isoflurane anesthesia using a rebound tonometer (TonoLab, Icare, Finland) within 5 min after the animal was knocked down. The IOP of both eyes was measured alternately for each mouse and repeated 10 times for each eye. Each of the 10 IOP values was derived by default settings of the TonoLab tonometer using six single IOP readings, whereby the highest and lowest IOP readings were excluded, and the remaining four readings were averaged. All IOP measurements were taken in the afternoon between 1 and 3 p.m. The IOP difference between eyes (ΔIOP) was calculated as the IOP of the injected eye minus that of the contralateral non-injected eye for each mouse (24), and was used for between-group comparisons at each time point to account for any physiological fluctuations on both eyes.

### Retinal Thickness Evaluation Using Optical Coherence Tomography

The optic nerve head regions of both eyes of all mice were scanned linearly using spectral-domain optical coherence tomography (Bioptigen, Leica Microsystems, Germany). The retinas were segmented using a custom-written software (25, 26) to determine the retinal nerve fiber layer (RNFL) thickness and total retinal thickness (TRT) along a sampling ring band of 0.234–0.324 mm radius centered on the optic nerve head (**Figure 2A**). RNFL thickness was defined as the average distance between the ILM and the ganglion cell layer within the ring band (**Figure 2B**). TRT was defined as the average distance between the internal limiting membrane (ILM) and the Bruch's membrane (BM) within the ring band (**Figure 2B**).

### Visual Function Assessment Using Optokinetic Behavioral Testing

Visual function was assessed using an OptoMotry optokinetic virtual-reality device (CerebralMechanics, Lethbridge, Alberta, Canada) in each awake, freely moving mouse to quantify the visual acuity (VA) and contrast sensitivity for both eyes over time. Each mouse was first habituated in the optokinetic testing device for 5 min. It has been shown that when one eye is closed, only motion in the temporal-to-nasal direction for the contralateral eye evokes the tracking response (27). Thus, the visual capabilities of each eye can be measured under binocular conditions simply by changing the direction of rotation of the visual presentation. Using the OptoMotry system, clockwise and anti-clockwise rotations were alternately presented to examine the left and right eyes, respectively in the same mouse during the same experimental session. The VA testing involved an increasing spatial frequency of sine wave grating starting from 0.042 cycles/degree (c/d) at a constant drift speed of 0.12°/s at 100% contrast. VA was identified as the highest spatial frequency that the mice could track. Contrast sensitivity testing involved a decreasing image contrast from 100% with a constant spatial frequency of 0.103 c/d. Contrast threshold (CT), which is the inverse of contrast sensitivity was determined from this testing, with a higher CT implicative of worse visual function. Both VA and CT measurements used a simple staircase method and ended when the optokinetic response could no longer be elicited.

### Statistical Analysis

Comparisons of IOP, ΔIOP, TRT, RNFL, VA and CT were conducted between experimental groups over time using two-way ANOVA and *post-hoc* Tukey's multiple comparisons correction tests. Prior to intracameral hydrogel injection in Group 3, comparisons of each parameter before and after oral scutellarin administrations were conducted using paired *t*-tests. Correlation analyses between cumulative ΔIOP and other parameters at the end time point, and linear regressions between time and TRT, RNFL, VA and CT were also conducted. All statistical analyses were conducted with GraphPad Prism version 9 (GraphPad Software Inc., La Jolla, CA, USA). Data were presented as mean ± standard error of mean (SEM). *P* < 0.05 was considered to be statistically significant.

## Results

### Chronic IOP Elevation Was Induced After Intracameral Hydrogel Injection With No Effects From Scutellarin

**Figure 1:**
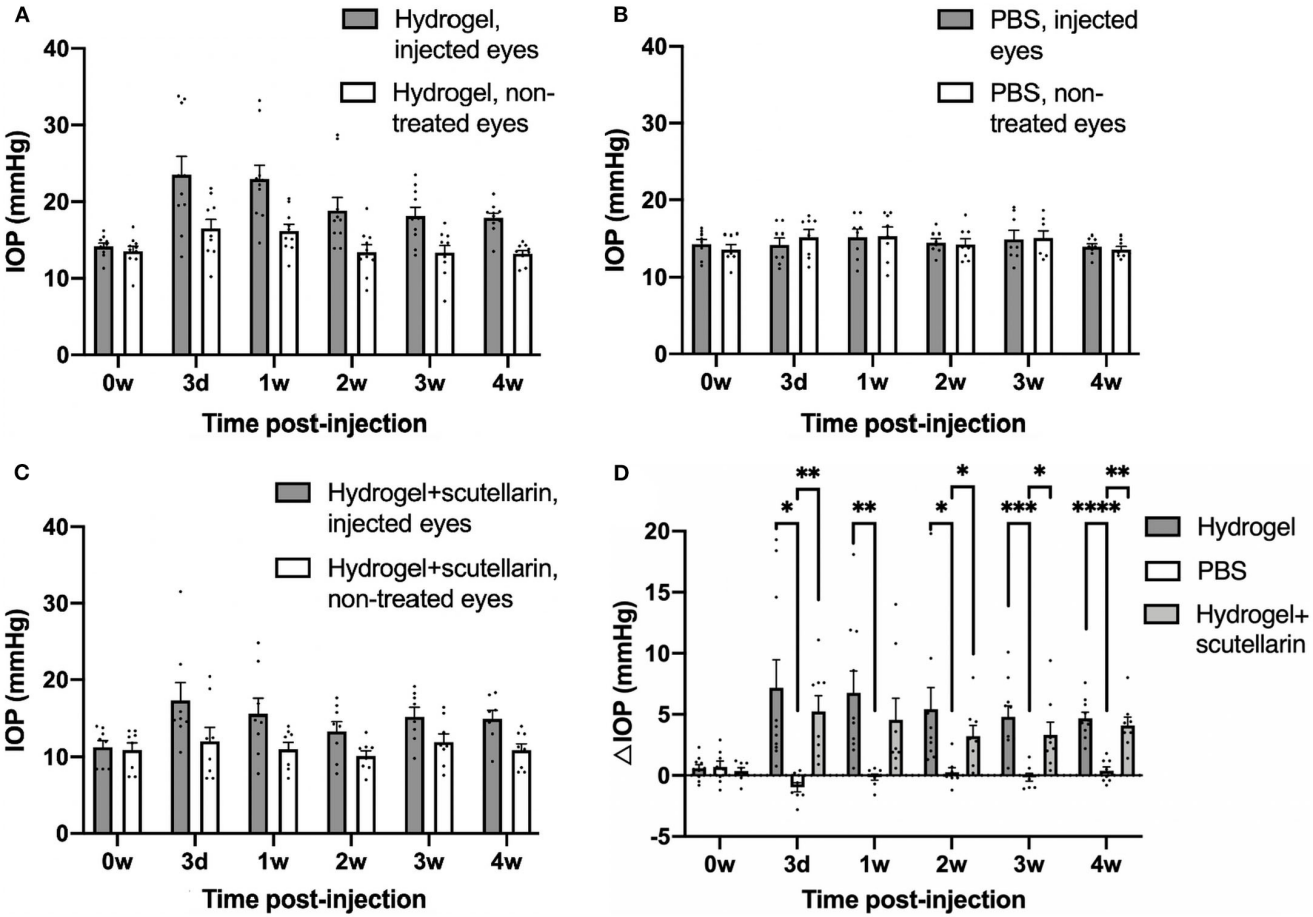
Intraocular pressure (IOP) profiles after unilateral intracameral injections in adult C57BL/6J mice. IOP values of both injected (gray bar) and contralateral, non-treated eyes (white bar) are shown in the hydrogel-only group (Group 1) **(A)**, phosphate-buffered saline (PBS) group (Group 2) **(B)** and hydrogel+scutellarin group (Group 3) **(C)**. Hydrogel-injected eyes in both Groups 1 and 3 **(A,C)** developed sustained elevation of IOP relative to the non-treated eyes (ANOVA, *p* < 0.05), while the PBS-injected eye in Group 2 did not differ in IOP levels from the non-treated eye (ANOVA, *p* > 0.05). **(D)** The inter-eye IOP difference (ΔIOP, injected minus non-injected eye) was significantly higher in both the hydrogel-only group (dark gray bar) and the hydrogel+scutellarin group (light gray bar) when compared to the PBS group (white bar), but was not significantly different between hydrogel-only and hydrogel+scutellarin groups. 0w: 0 week, pre-injection; 3d: 3 days post-injection; 1w to 4w: 1 week to 4 weeks post-injection. Data were represented as mean ± SEM. *Post-hoc* Tukey's multiple comparisons correction tests between groups in **(D)**: *p < 0.05, **p < 0.01, ***p < 0.001, ****p < 0.0001.

The hydrogel-injected eyes in Groups 1 (Figure 1A) and 3 (Figure 1C) had significantly higher IOP levels than the contralateral, non-treated eyes irrespective of scutellarin treatment (ANOVA, *p* < 0.05), while both eyes of the PBS group (Group 2, Figure 1B) had comparable IOP levels (ANOVA, *p* > 0.05). When comparing IOP of individual eyes, both the injected eyes and the contralateral non-treated eyes exhibited significant IOP differences between the three groups (ANOVA, *p* < 0.05). When comparing ΔIOP over time (Figure 1D), significant group differences were observed between the PBS group (Group 2) and the two hydrogel groups (Groups 1 and 3) after intracameral injection. No apparent ΔIOP difference was found between hydrogel-only group (Group 1) and hydrogel+scutellarin group (Group 3) at any experimental time point. No significant IOP difference was observed before and after oral scutellarin treatment prior to intracameral hydrogel injection in Group 3 (Supplementary Figure 1).

### Oral Scutellarin Treatment Reduced Retinal Thinning Under Hydrogel-Induced Chronic IOP Elevation

**Figure 2:**
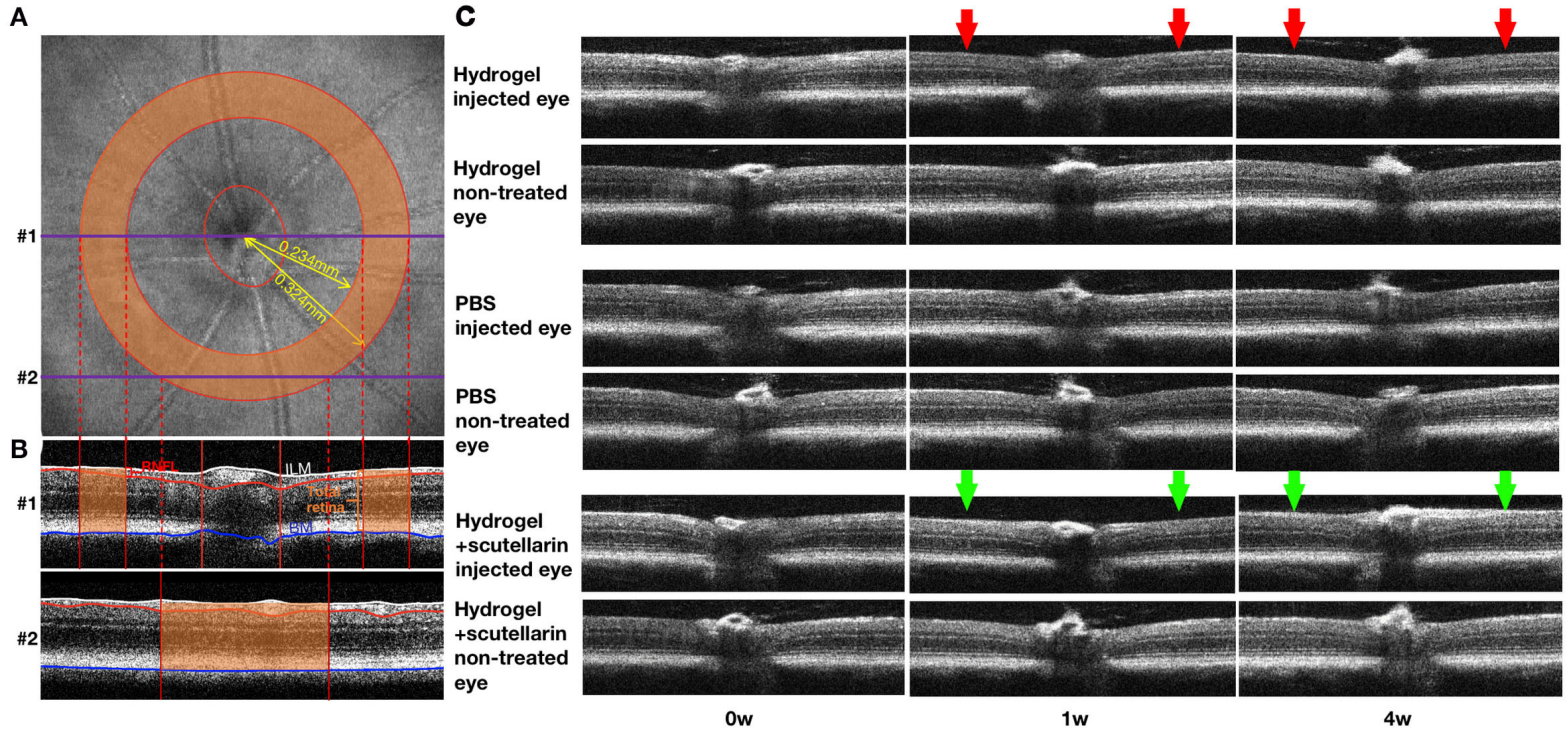
Optical coherence tomography of the mouse retina over time. **(A)** *en face* image showing the optic nerve head (ONH) and a sampling band (orange) of 0.234 to 0.324 mm radius centered on the ONH for measuring retinal nerve fiber layer (RNFL) thickness and total retinal thickness (TRT). **(B)** Two representative cross-sectional retinal B-scan images corresponding to the linear scans #1 (through the optic nerve) and #2 (around the optic nerve) in **(A)**. The RNFL (red bracket), inner limiting membrane (ILM; white line), and Bruch's membrane (BM; blue line) were outlined using a custom-written automated segmentation algorithm. RNFL thickness was defined as the distance between white and red lines, and TRT was defined as the distance between white and blue lines. Only the thicknesses within the orange sampling band were measured and averaged for each eye for further analyses. **(C)** Cross-sectional retinal images from both injected and contralateral, non-treated eyes of a representative mouse in each of the 3 groups at 0 week (0w; pre-injection), 1 week (1w) and 4 weeks (4w) after unilateral intracameral injection. Note the clear images indicating the transparency of the ocular media comprising the hydrogel. Note also the apparent inner retinal thinning in the injected eye of the hydrogel-only group (Group 1; red arrows), and less apparent retinal thinning in the hydrogel+scutellarin group (Group 3; green arrows). No obvious retinal change was observed in the injected eye of the PBS group and in the non-treated eyes of all three groups.

**Figure 3:**
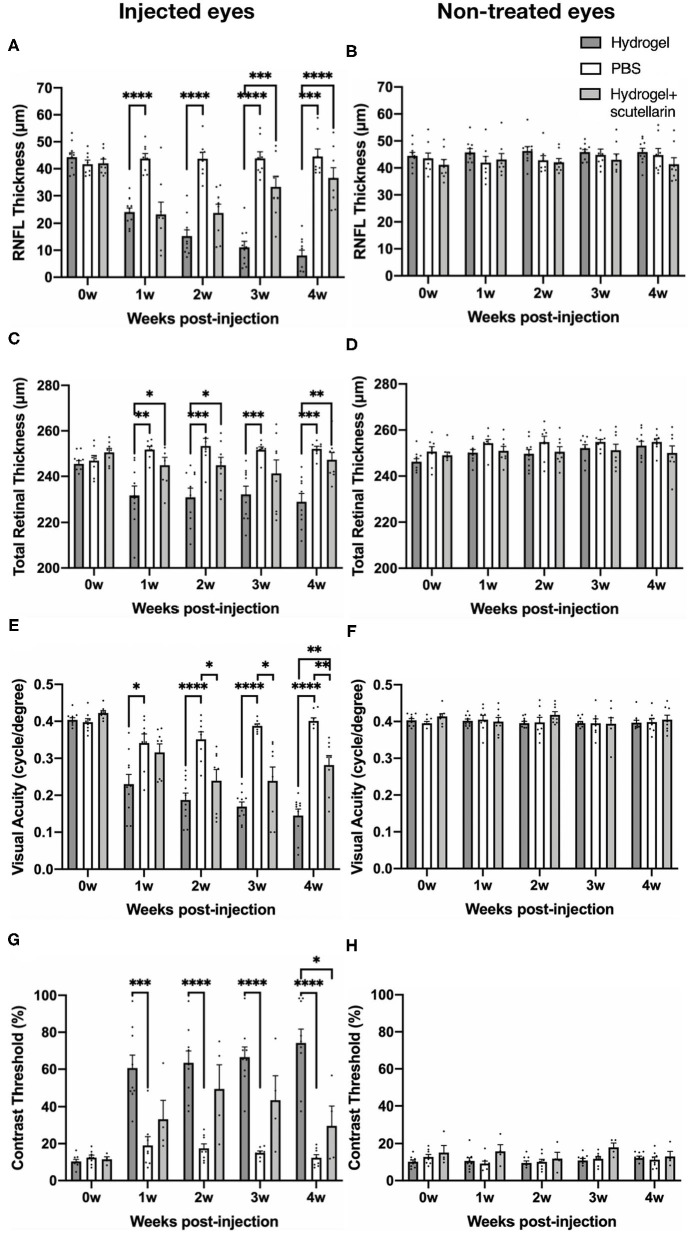
Quantitative analyses of retinal nerve fiber layer (RNFL) thickness, total retinal thickness (TRT), visual acuity, and contrast threshold in the injected (left column) and contralateral, non-treated eyes (right column) among the hydrogel-only group (Group 1, dark gray bar), phosphate-buffered saline (PBS) group (Group 2, white bar) and hydrogel+scutellarin group (Group 3, light gray bar). The scutellarin-treated, hydrogel-injected eyes in Group 3 showed less RNFL **(A)** and TRT **(C)** thinning, and reduced visual behavioral deficits **(E,G)** when compared to the untreated, hydrogel-injected eyes in Group 1. Such differences became more apparent toward the later stages of the experimental period. No significant change in RNFL thickness **(B)**, TRT **(D)**, visual acuity **(F)**, or contrast threshold **(H)** was observed in the non-treated eyes of all three groups or in the injected eye of the PBS group **(A,C,E,H)** (*p* > 0.05). 0w: 0 week, pre-injection; 1w to 4w: 1 week to 4 weeks post-injection. Data are represented as mean ± SEM. *Post-hoc* Tukey's multiple comparisons correction tests between groups: *p < 0.05, **p < 0.01, ***p < 0.001, ****p < 0.0001.

Figures 2A,B illustrate the regions of interest for measuring retinal thicknesses in both the *en face* image (Figure 2A) and the corresponding cross-sectional b-scan images in different line scans (Figure 2B). Figure 2C shows the cross-sectional retinal images from both injected and contralateral, non-treated eyes of a representative mouse in each of the 3 groups. The injected eye of the hydrogel-only group (Group 1) showed apparent inner retinal thinning over time, whereas less apparent retinal thinning was observed in the injected eye of the hydrogel+scutellarin group (Group 3). Quantitatively, relative to the PBS-injected eye in Group 2, RNFL thickness (Figure 3A) and TRT (Figure 3C) significantly decreased in the hydrogel-injected eye of Group 1 only without scutellarin treatment at each post-injection time point, whereas no statistically significant RNFL thickness or TRT change was observed in the hydrogel-injected eye of Group 3 after scutellarin treatment (*p* > 0.05). RNFL thickness and TRT of the injected eye in Group 3 were also significantly higher than those in Group 1 after hydrogel injection. In the hydrogel-only group, the cumulative ΔIOP was linearly correlated to RNFL thickness (R^2^ = 0.607, *p* = 0.008) and TRT (R^2^ = 0.401, *p* = 0.049) at 4 week post-injection. No apparent difference in RNFL thickness or TRT was found between the three groups in the injected eye at 0 week before intracameral injection (Figures 3A,C), or in the PBS-injected eye and non-treated eye over time (Figures 3B,D).

### Oral Scutellarin Treatment Ameliorated Visual Acuity Decrease and Contrast Threshold Increase Under Hydrogel-Induced Chronic IOP Elevation

The longitudinal profiles of VA and CT for each group are shown in Figures 3E–H, respectively. Relative to the PBS-injected eye in Group 2, VA of the hydrogel-injected eye in Group 1 progressively declined upon intracameral hydrogel injection, whereas upon oral scutellarin treatment in Group 3, VA of the hydrogel-injected eye apparently decreased more slowly than that of Group 1 without scutellarin treatment (Figure 3E; Supplementary Figure 2). VA of the injected eye in Group 3 was also significantly higher than that of Group 1 at 4 weeks after intracameral hydrogel injection (Figure 3E). CT of the hydrogel-injected eye in Group 1 was significantly higher than that of PBS-injected eye in Group 2 at each post-injection time point (Figure 3G). In contrast, CT of the hydrogel-injected eye in Group 3 was significantly lower than that in Group 1 at 4 weeks after intracameral injection (Figure 3G). Cumulative ΔIOP was not correlated to visual acuity or contrast threshold in any groups (*p* > 0.05). No apparent VA or CT change was observed in the PBS-injected eye in Group 2 throughout the experimental period (Figures 3E,G). No significant change in VA or CT was found in the non-treated eye between all three groups over time (Figures 3F,H).

## Discussion

In the current study, we applied oral scutellarin treatment to our recently developed experimental glaucoma model, and demonstrated the preservation of retinal structure and visual function by scutellarin via non-invasive assessments over time. This crosslinking hydrogel model produces chronic IOP elevation while preserves optical media clarity in the long term (22). The transparency of the bioinert hydrogel allows not only *in vivo* optical imaging of the retina but also longitudinal evaluation of awake optokinetic responses, both of which are important for understanding glaucomatous neurodegeneration and treatment effects. Here, given that scutellarin did not significantly affect IOP changes after intracameral hydrogel injection, our results may help expedite the development of neuroprotective therapies for glaucoma and other neurodegenerative diseases beyond IOP control.

Scutellarin, a multifunctional flavonoid, has been shown to exert protective effects on diabetes (28, 29), inflammation (30), tumors (31), and multiple organ diseases in the kidney, lung, and liver (32, 33). Within the central nervous system, scutellarin treatment displayed antioxidant and metal-chelating neuroprotective properties against Alzheimer's disease (34). Scutellarin has also been shown to alleviate hypoxia-induced cognitive impairment by promoting the proliferation and differentiation of neural stem cells in a mouse model (35). Chronic IOP elevation can induce ischemia and hypoxia of the optic nerve and its surrounding retinal tissue (36–38) causing retinal cell loss or neurodegeneration, which may lead to inner retinal thinning (39, 40). On the other hand, scutellarin can improve the axoplasmic flow and blood supply of the optic nerve (41), as well as the growth promotion and apoptosis inhibition of RGCs (42).

Scutellarin is also suggested to protect against a cascade of inflammatory events caused by glaucoma. In experimental glaucoma, models of chronic IOP elevation by intracameral microbead injection (43), acute IOP elevation by anterior chamber perfusion (21), and optic nerve crush (44) indicated the involvements of NLRP3 inflammasome activation during retina and optic nerve head damage, whereas pharmacological inhibition of NLRP3 (43) and inhibitor of Fas receptor (45) have been suggested as potential neuroprotective therapeutics in glaucoma. Given a recent experimental glaucoma study demonstrating increased expression of tumor necrosis factor-α, interleukin-1β (IL-1β) and IL-17A protein in the mouse retina after hydrogel-induced chronic IOP elevation (46), whereas scutellarin may inhibit the inflammatory processes of retinal neurodegeneration through the NLRP3 inflammasome signaling pathway, including NLRP3, apoptosis-associated speck-like protein containing a caspase recruitment domain, cleaved caspase-1, IL-1β, and IL-18 (21), it is possible that the mechanism of the reduced retinal thinning in our scutellarin-treated, hydrogel-injected eyes involved intervention of neuroinflammation via the NLRP3 inflammasome pathways. Scutellarin was also found to inhibit abnormally activated microglia and provide protection against neurodegeneration in the eye and the brain (21, 47, 48). Future research is warranted to examine if scutellarin may reduce inflammatory events in experimental chronic glaucoma (49, 50) and its associated damages to the retina and the optic nerve.

In terms of functional recovery, scutellarin has been shown to improve neurological functions in Alzheimer's disease mouse models (51) and in rats with cerebral ischemia (52, 53). Using multifocal electroretinogram, *Erigeron breviscapus* extract was also found to improve the impaired visual function of persistently elevated IOP in rats induced by episcleral vein cauterization (54). Our optokinetics results of ameliorated VA or CT aggravation in the scutellarin-treated, hydrogel-injected eyes further supported the role of oral scutellarin treatment in improving visual behavioral responses upon experimental glaucoma. While the exact mechanisms underlying the visual improvements remain to be elucidated, potential candidates may include mediation of the potassium (55) and calcium ions (56, 57). For example, flavonoid extracts of *Erigeron breviscapus* can suppress outward potassium currents in rat RGCs (55), which may help prevent RGC injury and vision loss caused by glaucoma. In addition, scutellarin may modulate intracellular calcium ion concentrations and voltage-gated calcium channels in the smooth muscle cells of vasculature (56), whereas systemic calcium ion antagonism can constantly improve visual field function in glaucoma patients (57). Further experimentations are necessary to determine the physiological basis of the observed visual recovery along with its linkage to the integrity of the neuroretina and visual pathway in more depth.

Scutellarin administration was found to exert protective effects in a dose-dependent manner (29, 58). We chose our current dosage of 300 mg/kg/day for 3 weeks with reference to similar flavonoid studies (59–61) in an attempt to maximize the delivery of scutellarin to the eye and the neural tissues. We have also attempted a low-dose pilot study (*n* = 5) using 50 mg/kg/day scutellarin treatment before formal experiments following a prior study (21), and did not observe obvious differences in visual behavioral responses from no scutellarin treatment (ANOVA *p* > 0.05, not shown). Therefore, the higher 300 mg/kg/day dose was chosen in the current formal experiments. Scutellarin was found to be minimally toxic or non-toxic in rodents up to 500 mg/kg/day (62), suggesting that our current dosage had a sufficient margin of safety for therapeutic use. Besides, we did not observe significant differences in body weight between the 3 groups in the current experiments (ANOVA *p* > 0.05, not shown). Scutellarin was suggested to protect visual function in glaucoma patients with controlled IOP at a lower oral dosage than the current study (20), whereas no side effect of scutellarin treatment on the eye has been illustrated in theory or reported in practice (63). At a high scutellarin dosage of more than 10 g/kg, systemic adverse effects including hypoactivity, loss of appetite, and asthenia were observed in mice which disappeared within 48 h (62), while another human study found rare occurrence of adverse drug reactions such as rash, chills and fever upon systemic scutellarin administration (63). Future studies are foreseen that expand upon the current findings to determine the dose-dependent effects of oral scutellarin treatment on vision preservation as well as potential adverse events in the eye.

There are several limitations for the experiments in the current study, one being the potential physiological fluctuations from anesthesia on IOP measurements. We expected that such effect was small, since we measured IOP soon after knocking the animals down from isoflurane induction, while all IOP measurements were taken in the same period between 1 pm and 3 pm to minimize diurnal variation. However, since not only the injected eyes but also the contralateral non-treated eyes exhibited significant IOP differences between the three groups, cautions should be noted when interpreting the IOP levels of individual eyes. Comparing the inter-eye IOP difference allowed us to evaluate the extent of IOP elevation induced by intracameral hydrogel injection more specifically and accurately, accounting for any physiological fluctuations on both eyes. Future studies may consider awake IOP measurements after training to further improve reliability and consistency (64).

With respect to the effects of oral scutellarin treatment on IOP, we compared IOP levels in Group 3 during the week of oral scutellarin treatment prior to hydrogel injection, and did not observe any significant differences before and after scutellarin treatment (Supplementary Figure 1). In addition, no significant difference in other parameters (i.e., RNFL thickness, TRT, VA and CT) was observed before and after oral scutellarin administration in Group 3 prior to hydrogel injection (Supplementary Figure 1). These findings suggest that 1 week of oral scutellarin treatment did not substantially affect the retinal structure or visual function in healthy adult mice. However, cautions should still be noted about the possibility that the slightly lower baseline IOP observed in the scutellarin-treated animals than the control groups may account for some of the protective effects observed in the experimental group. In addition, more evidence is needed to determine the possibility of secondary reduction of IOP elevation by oral scutellarin treatment after hydrogel injection.

Since scutellarin has been shown to exhibit unique pharmacokinetic behaviors in humans and animals that cannot be explained by the classical compartment model (65), how oral scutellarin administrations and the corresponding neurotherapeutic findings can be translated into the pharmacological activities and concentrations of scutellarin in the target brain tissues such as the visual system compartments remains unclear (63). To the best of our knowledge, the most relevant rodent brain drug distribution studies showed that 22% of orally and 29% of tail vein administered radiolabeled breviscapine (an extract mixture of *Erigeron breviscapus* with ≥90% scutellarin) reached the rat brain tissues when it was administered at a dose of 0.4 mg/kg (66). Despite the limited studies, rodents are considered as a preferred animal model for translating scutellarin studies to humans, as the model better mimics the pharmacokinetic behaviors and bioavailability of scutellarin in humans relative to other species (63). In addition, upon oral scutellarin administration, substantial amounts of scutellarein (i.e., the aglycone form of scutellarin) are present in the blood of both humans and rodents (65). In future studies, plasma measurements of scutellarin and scutellarein, as well as local examination of the NLRP3 inflammasome signaling pathway may help examine more specifically the therapeutic effects, pharmacokinetics, and pharmacological activity of the drug. Since scutellarin has been reported with a relatively low bioavailability upon oral administration (65), solutions can also be exploited to enhance oral delivery efficacy of scutellarin to glaucoma models or patients, such as the use of vitamin B12 derivatives-modified nanoparticles (67).

Given that the course of the disease deterioration in glaucoma is chronic and endures for years, further studies with higher frequencies of experimental measurements, longer periods of experimental follow-ups and large sample sizes can help to determine the safety and effectiveness of long-term scutellarin oral treatment, while histological and immunohistochemical studies would help further investigate the mechanisms underlying the *in vivo* findings observed in the current study. Future studies may also include electroretinography to provide additional functional endpoints to the visual behavioral optokinetic assessments. Future experimental designs can also consider post-IOP elevation treatment only or combined IOP lowering and scutellarin treatment for stronger clinical relevance. Overall, this preliminary but potentially important study demonstrated the use of a non-invasive measuring platform to examine retinal thinning and visual behavioral deficits after hydrogel-induced chronic IOP elevation, as well as the positive role that scutellarin played on retinal structure and visual function under chronic experimental glaucoma. Scutellarin may be a possible candidate as a novel neurotherapeutic agent for glaucoma treatment beyond IOP control.

## Data Availability Statement

The original contributions presented in the study are included in the article/Supplementary Material, further inquiries can be directed to the corresponding author/s.

## Ethics Statement

The animal study was reviewed and approved by NYU Grossman School of Medicine's Institutional Animal Care and Use Committee (IACUC).

## Author Contributions

JZ, CL, GW, JSS, WW, and KC: study conception and design. JZ, AS, KA, VA, JRS, EY, and YYu: data collection. JZ, KA, EY, YYa, HI, and KC: data analysis and interpretation. JZ, JRS, EY, YYa, HI, GW, JSS, and KC: manuscript writing. All authors have read and approved the final manuscript.

## Conflict of Interest

YYu and CL have patents filed related to this study (Induction of chronic elevation of intraocular pressure with intracameral cross-linking hydrogel US 20150250815). YYu was employed by the company Pleryon Therapeutics Limited. The remaining authors declare that the research was conducted in the absence of any commercial or financial relationships that could be construed as a potential conflict of interest.

## Publisher's Note

All claims expressed in this article are solely those of the authors and do not necessarily represent those of their affiliated organizations, or those of the publisher, the editors and the reviewers. Any product that may be evaluated in this article, or claim that may be made by its manufacturer, is not guaranteed or endorsed by the publisher.
