# Supplementary figures and images for: Oral Scutellarin Treatment Ameliorates Retinal Thinning and Visual Deficits in Experimental Glaucoma
Source: Front Med (Lausanne). 2021 Aug 3;8:681169. doi: 10.3389/fmed.2021.681169 (PMC8369066; doi:10.3389/fmed.2021.681169)

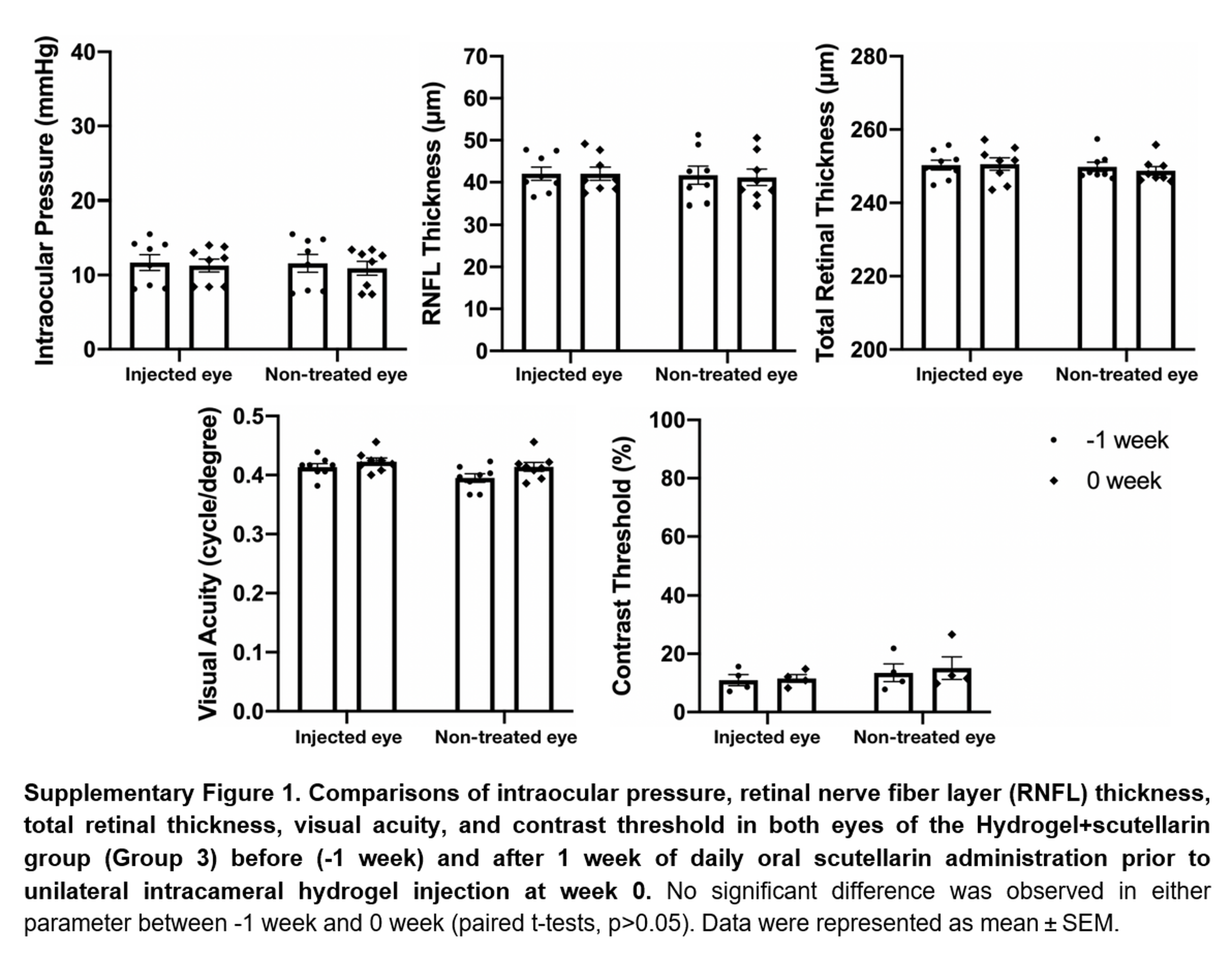

Supplement: Supplementary file 1 [file Image_1.TIF]

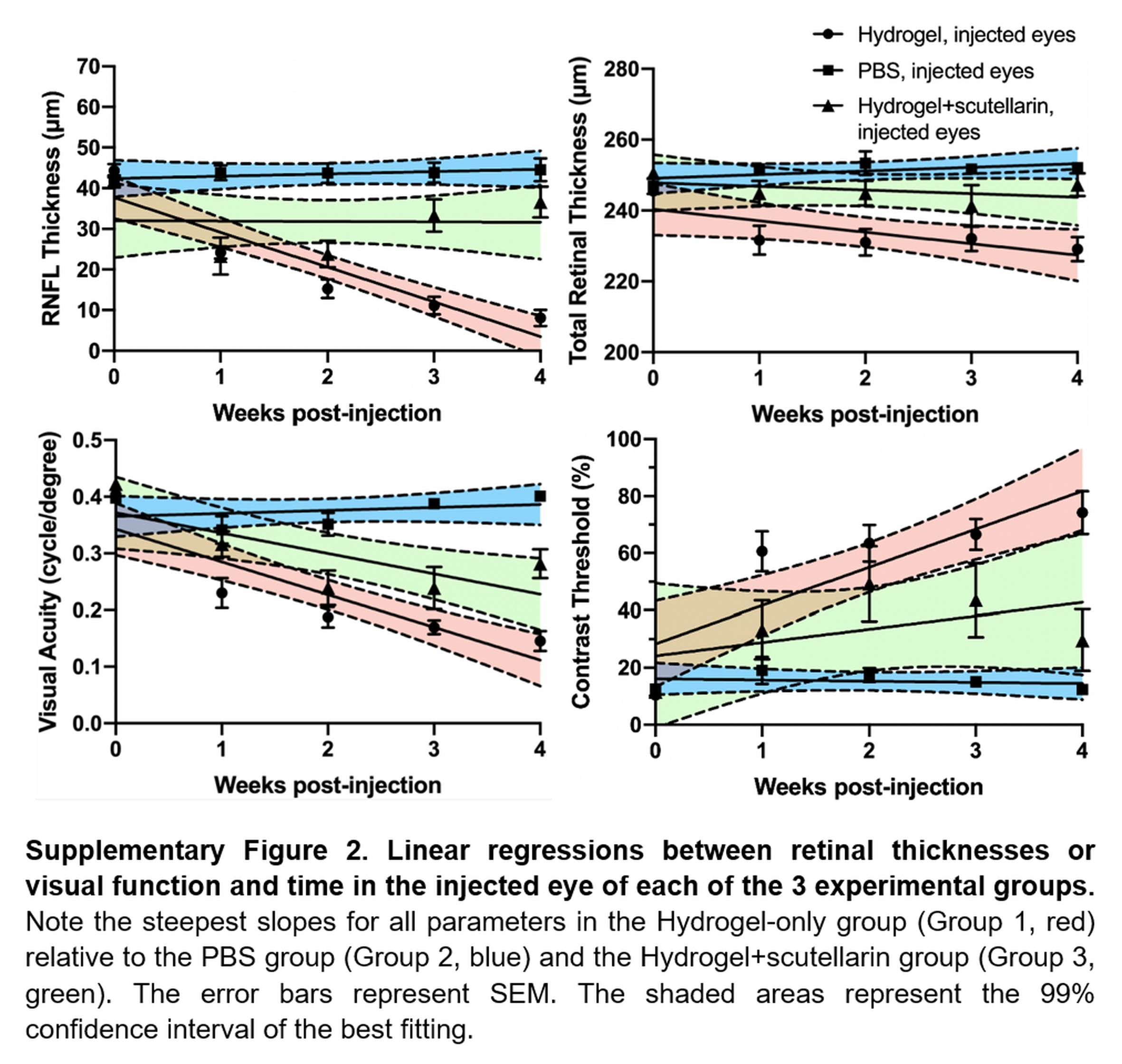

Supplement: Supplementary file 2 [file Image_2.TIF]
